# Blood Transfusion Delay and Outcome in County Hospitals in Kenya

**DOI:** 10.4269/ajtmh.16-0735

**Published:** 2017-02-08

**Authors:** Julius Thomas, Philip Ayieko, Morris Ogero, Susan Gachau, Boniface Makone, Wycliffe Nyachiro, George Mbevi, Mercy Chepkirui, Lucas Malla, Jacquie Oliwa, Grace Irimu, Mike English

**Affiliations:** 1 KEMRI-Wellcome Trust Research Programme, Nairobi, Kenya.; 2 Nuffield Department of Medicine, University of Oxford, Oxford, United Kingdom.; 3 Department of Paediatrics and Child Health, University of Nairobi, Nairobi, Kenya.

## Abstract

Severe anemia is a leading indication for blood transfusion and a major cause of hospital admission and mortality in African children. Failure to initiate blood transfusion rapidly enough contributes to anemia deaths in sub-Saharan Africa. This article examines delays in accessing blood and outcomes in transfused children in Kenyan hospitals. Children admitted with nonsurgical conditions in 10 Kenyan county hospitals participating in the Clinical Information Network who had blood transfusion ordered from September 2013 to March 2016 were studied. The delay in blood transfusion was calculated from the date when blood transfusion was prescribed to date of actual transfusion. Five percent (2,875/53,174) of admissions had blood transfusion ordered. Approximately half (45%, 1,295/2,875) of children who had blood transfusion ordered at admission had a documented hemoglobin < 5 g/dl and 36% (2,232/6,198) of all children admitted with a diagnosis of anemia were reported to have hemoglobin < 5 g/dL. Of all the ordered transfusions, 82% were administered and documented in clinical records, and three-quarters of these (75%, 1,760/2,352) were given on the same day as ordered but these proportions varied from 71% to 100% across the 10 hospitals. Children who had a transfusion ordered but did not receive the prescribed transfusion had a mortality of 20%, compared with 12% among those transfused. Malaria-associated anemia remains the leading indication for blood transfusion in acute childhood illness admissions. Delays in transfusion are common and associated with poor outcomes. Variance in delay across hospitals may be a useful indicator of health system performance.

## Background

Severe anemia mostly attributable to malaria is a leading indication for blood transfusion and an important cause of hospital admission and mortality in African children.^1^–^6^ The World Health Organization (WHO) has provided guidelines to inform clinical decisions on transfusion linked to hemoglobin level.^7^–^10^ On the basis of these guidelines, urgent blood transfusion is recommended for children with severe malaria and hemoglobin level < 5 g/dL if they are in respiratory distress.^11^–^13^ Although these guidelines can be lifesaving,^14^–^17^ adherence to its recommendations is poor,^18^ and when used, there still might be need for retransfusion.

Despite clear transfusion policies and clinical guideline recommendations, the inability to initiate blood transfusion rapidly enough to save lives has been identified as a contributing factor to anemia deaths in sub-Saharan Africa.^14^,^19^ Considerable investments have been made in the blood transfusion services in Kenya over recent years to provide regional blood banks and improved screening to reduce possible risk of acquiring transfusion-transmitted infections.^12^,^20^,^21^ Prior to nationwide efforts to improve transfusion services, significant health system challenges existed in availability and delivery of blood for transfusion,^21^–^24^ and this often made urgent transfusion almost impossible.^21^ At the patient level, prior audits of care for malaria-associated anemia admissions have reported problems with ordering and documentation of basic laboratory investigations. Furthermore, hemoglobin measurement and evidence of inappropriate transfusion practices have also been observed,^16^,^21^,^24^ and such audits, however, are often limited in scope over time or place.

This study sought to examine the ability of clinicians to access blood when needed in Kenya after efforts to improve supply by exploring evidence of any delays in transfusion in Kenyan hospitals. We also examined use of hemoglobin measurement as a guide to transfusion and the outcomes of children with anemia requiring blood transfusion according to their transfusion experience. Importantly, we did this for a set of hospitals and over a prolonged period. Such measures of transfusion delay are potentially useful indicators of a health system's ability to support quality care.

## Methods

### Study setting.

The study was nested within the Clinical Information Network (CIN). The CIN is a Kenyan collaborative initiative among the KEMRI-Wellcome Trust Research Program, The Ministry of Health, The Kenya Pediatric Association, and a set of 14 Kenyan county referral hospitals. The CIN, as a pragmatically common system for collecting patient level data from all pediatric admissions, aims to use the information to improve health care practices. Characteristics of these 14 hospitals and their admission populations have been reported elsewhere.^25^ The hospitals are located in 11 counties that were first identified with the Kenyan Ministry of Health to represent different settings, including varying malaria transmission settings, while being feasible for inclusion in the CIN.

During hospital selection, public hospitals offering first referral level care within these counties that had at least 1,000 pediatric admissions yearly were considered eligible. This resulted in two hospitals being selected from one large urban county. In addition, two facilities in the same county town were selected as it was feasible to collect data from both. Therefore, a total of 14 hospitals joined CIN. Of these hospitals, four (H4, H5, H9, and H14) were located in highland locations and had no cases of blood transfusion in the study population and over the study period and were therefore excluded from this analysis.

### Study population.

The study population comprised pediatric nonsurgical admissions aged 1 month and above who were admitted to 10 CIN hospitals. The subset of children in this population with a blood transfusion ordered constituted the subgroup of focus. The period of study was from September 2013 to March 2016.

### Data collection.

For all the pediatric ward admissions across CIN, data were collected and managed according to procedures described in full elsewhere.^26^ In brief, CIN hospitals implement standardized pediatric admission record and discharge forms that are part of the hospital medical record^27^ that together with the hospital's treatment sheets and laboratory ordering forms are completed by clinicians on duty. A clerical assistant retrospectively reviews all records on discharge and enters data directly into an electronic tool, REDCap (Vanderbilt University, Nashville, TN),^26^ that has inbuilt range and consistency checks. Data entry is guided by detailed standard operating procedures. Apart from clinical notes, data are also checked from laboratory records. Clerks receive initial training and refresher training by a data coordinator who visits sites approximately bimonthly and is available to answer any queries by phone. After the entry, data are checked on-site using automated routine scripts and errors are corrected before submission to a central server at the end of each day. The data are checked again for errors by the research team after the clerk has synchronized data to the server with any further queries being communicated to the clerk.^26^

In Kenyan health facilities, blood transfusion is mostly performed to treat malaria-associated anemia, severe acute malnutrition, and sickle cell anemia patients and to replenish massive blood loss in the course of childbirth, accident, or surgery.^14^,^28^ Our analysis focused on inpatient pediatric wards with information collected on admissions' symptoms and signs, orders for blood transfusion, date blood transfusion prescribed, date blood transfusion given, hemoglobin results, admission diagnoses, and final outcome at discharge. In these settings, transfusion orders are for whole blood or much less commonly packed red cell. Other blood components (platelets or plasma) are not routinely available.

### Data analysis.

Data were analyzed using R Statistical software, Version 3.1.3 (Robert Gentleman and Ross Ihaka, University of Auckland, New Zealand). Descriptive statistics were used to summarize patient demographic characteristics, represented with means, counts, and proportions where appropriate. The delay in blood transfusion was calculated from the date when transfusion was prescribed to date of actual transfusion. A simple graphical approach was used to illustrate the pattern of delay for each hospital. To determine the effects of delayed transfusion among the children, we retrospectively classified them as not transfused or transfused and then as transfused with no delay (transfusion on the same day as the order) or with delay (transfusion one or more days after the order).

Logistic regression models were used to evaluate risk of death among children with blood transfusion ordered at admission. After testing for possible interactions between risk of death predictors, the association of risk factors with death of variables with a *P* value < 0.05 in univariate analysis were included in a multivariable logistic regression analysis. Hospitals were treated as fixed effects to explore their potential association with death.

### Ethics approval.

KEMRI Scientific and Ethical Review Committee approved the CIN study. The Kenya Ministry of Health gave permission for this work to be done but no individual patient consent was obtained.

## Results

### Patient characteristics.

During the study period between September 2013 and March 2016, a total of 53,174 children were admitted to the 10 CIN hospitals. The mean age of the study population was 33.6 months (standard deviation = 34.6), and 54% (28,943/53,174) of all the pediatric admissions were male (Table 1). Overall, 41% (21,628/53,174) of all children admitted to these hospitals had malaria, and 12% (6,198/53,174) had anemia, of which 68% (4,198/6,198) had severe anemia. Two percent (1,270/53,174) of admissions were diagnosed with sickle cell disease. Of all the anemia cases, 56% (3,487/6,198) had a hemoglobin measurement taken, of which 64% (2,232/3,487) had hemoglobin levels < 5 g/dL.

### Blood transfusion practices.

**Figure 1.**
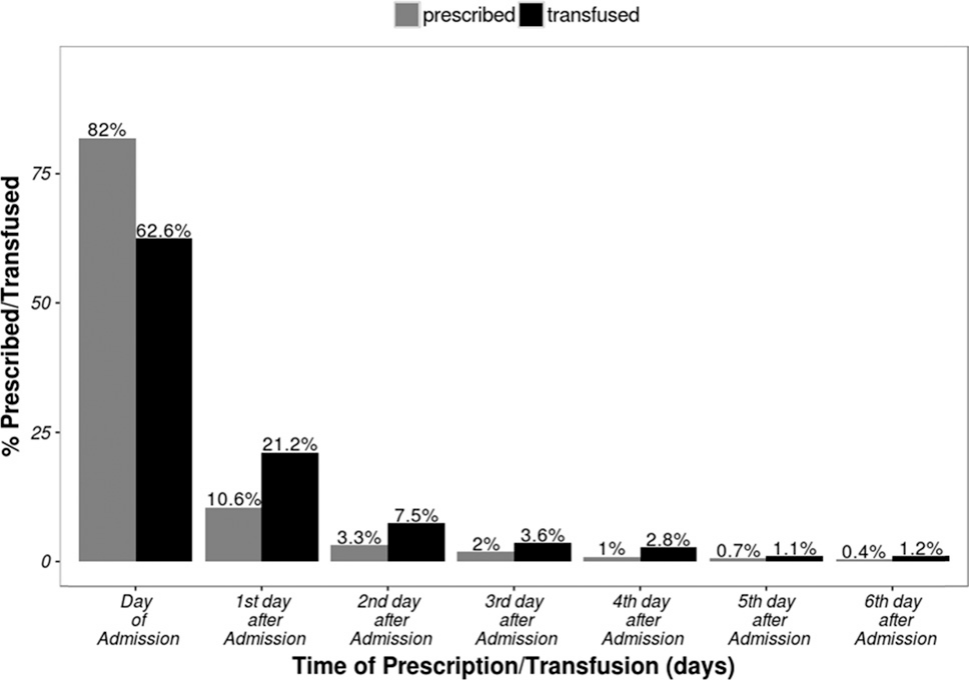
Time between prescription and transfusion from the day of admission for all children who had blood prescribed in 10 Clinical Information Network hospitals.

Five percent (2,875/53,174) of admissions had blood transfusion ordered. In most cases, the diagnoses associated with the order for blood transfusion were anemia 78% (2,240/2,875) and malaria 73% (2,094/2,875) (Table 1). Less than 10% of the children for whom blood transfusion was ordered had severe acute malnutrition (8%, 226/2,875) or both malaria and sickle cell disease (5%, 149/2,875). Approximately half (45%, 1,295/2,875) of children who had blood transfusion ordered at admission had a documented hemoglobin < 5 g/dL and 36% (2,232/6,198) of all children admitted with a diagnosis of anemia were reported to have hemoglobin < 5 g/dL. Of all the ordered transfusions, 82% (2,352/2,875) were documented to have been given, and three-quarters of these (75%, 1,760/2,352) were given on the day they were ordered (Figure 1

).

**Figure 2.**
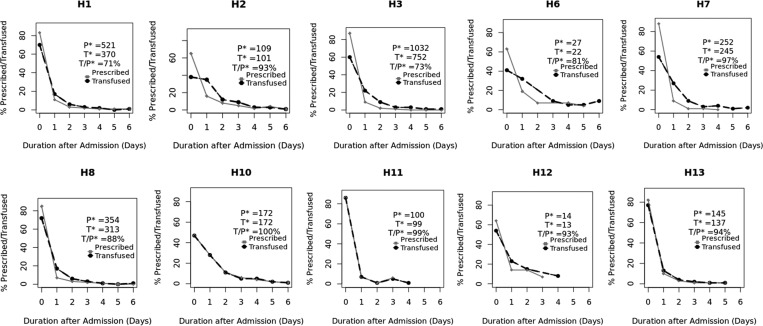
Hospital-specific charts of time of prescription order and time to transfusion in days. P* = total cases prescribed for blood transfusion for the specific hospital during the period, T* = total cases transfused for the specific hospital during the period, T/P* = percentage of the prescribed cases that were transfused for the specific hospital during the period.

The number of blood transfusions prescribed within hospitals during the period ranged from 14 to 1,032 (Figure 2

). Blood transfusion prescriptions that were actually administered per hospital ranged between 71% and 100%. Exploring the delay between transfusion orders and the blood being given for individual hospitals suggest important differences (Figure 2). In three of the 10 hospitals, there were no apparent delays in administering blood transfusion, represented by complete overlap of curves for prescription and administration of blood (H10, H11, and H13). In three hospitals, delays appeared infrequently (curves close together, H1, H8, and H12), whereas a final group (four of 10) had higher frequency of delays (curves more widely apart H2, H3, H6, and H7) (Figure 2).

### Mortality.

**Figure 3.**
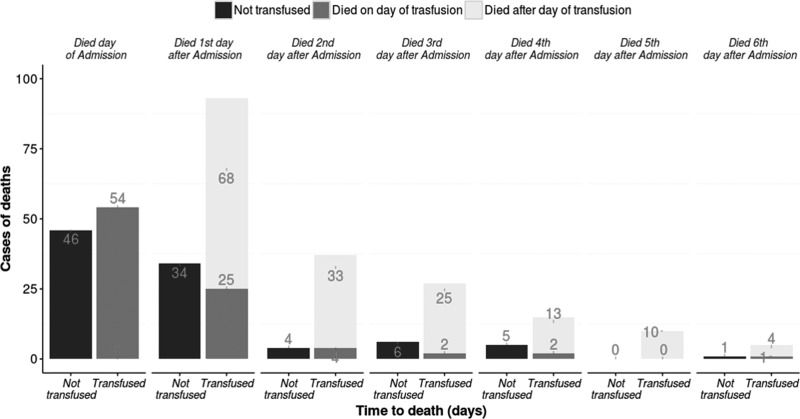
Timing of deaths occurring in children prescribed blood transfusion.

Seven percent (3,486/53,174) of admissions in the 10 hospitals died, and 13% (381/2,875) of these deaths occurred in children for whom a transfusion was ordered. Among the patients with blood transfusion prescribed (*N* = 2,875), the group which was not transfused witnessed the highest mortality of 20% (105/523) compared with 12% (276/2,352) among those transfused. Most deaths of children for whom blood was ordered but not given occurred on the admission day and the following day (*N* = 46 and 34, respectively, Figure 3

). For those who received a transfusion, a considerable number of deaths still occurred on the day of admission and transfusion (*N* = 54). However, a large number of children transfused on the admission day died on the following day despite this transfusion (*N* = 68). In total, there were 153 deaths among children who were transfused that occurred 1 day or more after transfusion was provided as an intervention (Figure 3).

Among children for whom a blood transfusion was ordered, mortality was lower in those with a diagnosis of malaria (adjusted odds ratio [OR] = 0.4, 95% confidence interval [CI] = 0.33–0.50) or anemia (OR = 0.41, 95% CI = 0.33–0.52) or findings of severe pallor (OR = 0.65, 95% CI = 0.53–0.81). The following variables were associated with an increased odds of death: malnutrition (OR = 2.56, 95% CI = 1.74–3.75); presence of lower chest-wall indrawing (OR = 2.81, 95% CI = 1.99–3.93); altered consciousness represented either by response to verbal stimuli (alert, verbal response, pain response and unresponsive [AVPU] = verbal response [V], OR = 2.64, 95% CI = 1.43–4.7) or responding only to pain or unresponsive (AVPU < *P*, OR = 3.81, 95% CI = 2.66–5.43); grunting (OR = 2.05, 95% CI = 1.35–3.1); and severe jaundice (OR = 3.12, 95% CI = 1.66–5.61).

In the model adjusting for such features of severe illness and compared with children who had a transfusion ordered and given on the same day, those with a delay in receiving blood (≥ 1 day after prescription) had lower mortality (OR = 0.58, 95% CI = 0.38–0.87), whereas those not transfused had higher mortality (OR = 1.8, 95% CI = 1.3–2.49) (Table 2).

There was also a suggestion of significant variation in outcomes among those ordered for transfusion across the hospitals after adjusting for patient-level risk factors (Table 2).

## Discussion

Other studies have examined outcomes and treatments of children with severe anemia^29^ and those requiring transfusion.^4^,^30^ However, few studies have examined data from multiple sites over prolonged periods of time. The need for blood is highly variable. In the CIN, 29% (4/14) hospitals did not report a transfusion prescription among pediatric admissions aged more than 1 month without a surgical diagnosis or burns over a period of 2 years. All these hospitals were in highland areas with almost no malaria.^25^ In the other 10 hospitals, 5% (2,875/53,174) of all infant and child medical admissions were felt to need blood. However, two hospitals H1 (521) and H3 (1,032) located in areas with considerable malaria transmission together accounted for over 50% of all transfusions ordered and given. Health systems, and particularly their blood transfusion services, obviously need to be organized to deal with such heterogeneity to ensure timely transfusion is possible.

Our data look moderately encouraging in terms of the ability of existing systems to provide blood when it is requested. Generally, eight of 10 (82%, 2,352/2,875) of all the orders for transfusion at admission result in blood administration, with 75% (1,760/2,352) of these children being transfused on the same day. Thus, overall 61% of all transfusions ordered were on the same day as the prescription as recommended in WHO and national transfusion guidelines.^12^,^24^ However, the proportion of transfusion orders that were completed varied across hospitals from 71% (H1) to 100% (H10 and H11) as did the frequency of delays in blood transfusion (Figure 2). In three hospitals with moderate numbers of transfusions, all of which are in urban settings with lower malaria endemicity, delays appear very infrequent (H10, H11, and H13; Figure 1). Proximity to regional blood bank centers may explain this scenario. In other hospitals (H2, H6, and H7; Figure 1), delays are more common and two of these hospitals are located in highly endemic malaria regions (H2 and H7). These hospitals perhaps experience more demand for blood than their supplies can support, whereas lack of proximity to a regional blood banking service could contribute to the delays observed in H6. Such data that point perhaps to localized challenges in the blood transfusion system are uncommon from low-resource settings.

The main reason for ensuring that blood is readily available for immediate transfusion when it is needed is to reduce mortality. Severe anemia, particularly related to malaria and respiratory distress, is associated with a high risk of mortality early in admission.^14^ In our findings, mortality was lower in those with a diagnosis of malaria and anemia. Possible reasons for this relatively lower mortality are the higher risks associated with sickle cell disease and severe malnutrition associated with severe anemia in these settings or that children with anemia may have had a previous recent episode of malaria now complicated by a severe bacterial infection including cases of septicemia that are rarely diagnosed in Kenyan hospitals that lack facilities for routine blood culture.

Overall mortality in children for whom a transfusion was ordered was 13% (381/2,875), among those transfused it was 12% (276/2,352), and among those not transfused it was 20% (105/523). An association of failure to transfuse with mortality was supported by analyses adjusting for other risk factors including signs of severe illness. Improved access to blood would therefore be expected to save further lives. In multivariable analyses, an association with reduced mortality was seen for children whose transfusion was not given on the day it was ordered. A possible explanation is that health workers make particular efforts to get blood for the sickest children and are happier to accept a delay in children who are less unwell. This would create bias by indication if it is the case. Unfortunately, we are unable to explore the possibility of this bias but anecdotally it is the case that greater efforts are made to obtain blood for the sickest children and that where blood supplies are scarce, the sickest children are prioritized for transfusion. Perhaps, an unexpected finding is the apparently large number of deaths (153/381 or 40% of all deaths in those ordered transfusion) that occurred after receipt of a transfusion. This too may represent an indication bias, that is, the sickest children are most likely to be prioritized for transfusion but die for reasons other than their anemia. It is also possible that the most profoundly anemic are transfused and that the first transfusion (typically standardized to 20 mL/kg of whole blood) does not adequately address the severity of the anemia as has been reported elsewhere.^18^

There is a paucity of data pertaining to key health indices (e.g., blood utilization) in low-income countries. The more system-wide District Health Information System (DHIS, version 2)^31^ that is commonly used across Africa is typically implemented to focus on reporting of workloads, morbidity, and mortality rather than elements of service provision. We have established as a partnership between the Ministry of Health, hospitals, the Kenyan Pediatric Association, and a research group to develop a CIN. Using relatively low-cost strategies to support much improved data collection,^26^ we have been able to provide a better understanding of the variability in presentations, treatments, and outcomes for admitted children.^25^ Here, we use the same system to explore access to a lifesaving intervention, blood transfusion. The simple metrics focused on transfusion we report could be a useful starting point for a wider system of monitoring the effectiveness of the blood transfusion service, particularly if data from other clinical areas (e.g., maternity and surgical departments) were collected in a similar fashion. More broadly, we believe there are considerable potential benefits from establishing improved, multipurpose information systems that can support evaluation of hospitals' services. These may initially take the form of specific networks, perhaps of sentinel sites, while lessons are learned on how to scale up such approaches and integrate them with the wider health information system.

This study has a number of limitations. Some hospitals had very few ordered transfusions (e.g., H6 and H12). We include data from these hospitals for completeness and to illustrate variability in need for blood. Indications for blood transfusion may, however, be quite different in such settings. It is also difficult to gauge appropriateness of blood use or associations with blood transfusion delays without further details on cases and hospitals that are difficult to acquire from discharge case record review. Thus, further information on hospitals' bed number and occupancy, spectrum of pathology, and clinical practice conditions would enable a more complete examination of the appropriateness of blood use and variability in transfusion practice (by physician, institution, or both). We were also not able to capture any data on the origins of delays in transfusion once ordered. Possible explanations include shortfalls in provision (i.e., strained inventories) and logistical/supply chain challenges. In the future, the transfusion indicators we report could be used together with targeted audits or linked to local quality improvement cycles to identify and address challenges locally linking system surveillance with measures to address local challenges.

## Conclusion

Malaria remains the leading diagnosis associated with the need for blood transfusion in acute childhood illnesses. There still exist significant delays between blood transfusion orders and actual transfusion in some hospitals and such delays are associated with poorer outcomes. The mortality among children ordered blood transfusion remains high pointing to the need for both better prevention and systematic identification of barriers to rapid transfusion when this is needed. Understanding whether blood transfusion services and outcomes are improving will require continued collection of data to allow basic metrics of service provision to be reported. The Clinical Information network may provide one means to support transition to data informed health system society.

Data for this report are under the primary jurisdiction of the Ministry of Health in Kenya. Enquiries about using the data can be made to the KEMRI-Wellcome Trust Research Programme Data Governance Committee.

## Figures and Tables

**Table 1:** Characteristics of children admitted in 10 CIN hospitals from September 2013 to March 2016

Characteristics	*N* (%)
Demographic characteristics of children in hospitals	
Mean age in months	33.6
Proportion of males all admissions	28,943/53,174 (54%)
Summary of transfusion practices	
Proportion of admission with	
Malaria	21,628/53,174 (41%)
Anemia*	6,198/53,174 (12%)
Severe anemia	4,198/6,198 (68%)
SCD	1,270/53,174 (2%)
Proportion of anemia cases with	
Hb measured	3,487/6,198 (56%)
Hb < 5 g/dL	2,232/6,198 (36%)
Hb measured where Hb < 5 g/dL	2,232/3,487 (64%)
Proportion of admissions with transfusion ordered	2,875/53,174 (5%)
Proportion of ordered transfusions that had a diagnosis of	
Anemia	2,240/2,875 (78%)
Malaria	2,094/2,875 (73%)
SCD	293/2,875 (10%)
Malnutrition	226/2,875 (8%)
Both malaria and SCD	149/2,875 (5%)
Other	157/2,875 (5%)
Proportion of ordered transfusions for whom Hb known to be < 5 g/dL	1,295/2,875 (45%)
Proportion of ordered transfusions given	2,352/2,875 (82%)
Proportion of transfusions given that were on the same day as the order	1,760/2,352 (75%)
Mortality	
Mortality among the study population	3,486/53,174 (7%)
Mortality among cases ordered for transfusion	381/2,875 (13%)
Mortality among cases transfused	276/2,352 (12%)
Mortality among cases not transfused	105/523 (20%)

CIN = Clinical Information Network; Hb = hemoglobin; SCD = sickle cell disease.

* All cases of anemia, inclusive of severe anemia cases.

**Table 2:** Predictors of the risk of death in children ordered blood transfusion at admission

Variable	Unadjusted OR (95% CI)			*N*	Adjusted OR (95% CI)	
	*N*	OR	*P* value		OR	*P* value
Transfusion ordered	2,875					
Age (years) level	2,557			2,239		
Less than 1 year		1 (Reference)				
1–4 years		0.45 (0.34–0.58)	< 0.001		0.73 (0.52–1.02)	0.066
Years ≥ 5		0.43 (0.32–0.57)	< 0.001		1.02 (0.70–1.49)	0.928
Anemia	2,040	0.41 (0.33–0.52)	< 0.001		0.63 (0.47–0.86)	0.004
Malaria	2,094	0.40 (0.33–0.50)	< 0.001		0.51 (0.37–0.71)	< 0.001
Malnutrition	226	3.59 (2.65–4.82)	< 0.001		2.56 (1.74–3.75)	< 0.001
Sickle cell disease	293	0.54 (0.35–0.81)	0.005		0.66 (0.38–1.10)	0.127
Severe pallor	1,831	0.65 (0.53–0.81)	< 0.001		0.66 (0.49–0.89)	0.007
Indrawing	427	5.65 (4.46–7.16)	< 0.001		2.81 (1.99–3.93)	< 0.001
Grunting	200	6.01 (4.43–8.13)	< 0.001		2.05 (1.35–3.10)	0.001
AVPU level	2,726			2,578		
Alert		1 (Reference)				
Verbal response		2.81 (1.72–4.46)	< 0.001		2.64 (1.43–4.70)	0.001
Pain response and unresponsive		4.73 (3.54–6.30)	< 0.001		3.81 (2.66–5.43)	< 0.001
Jaundice level	2,783			2,691		
None		1 (Reference)				
+ (mild/moderate)		0.60 (0.39–0.88)	0.011		1.01 (0.62–1.61)	0.964
+ + + (severe)		1.57 (0.92–2.55)	0.081		3.12 (1.66–5.61)	0.001
Transfusion given level						
Given same day		1 (Reference)				
Given different day		0.46 (0.32–0.64)	< 0.001		0.58 (0.38–0.87)	0.011
Not transfused		1.52 (1.18–1.94)	0.001		1.80 (1.30–2.49)	0.001
Hospital level						
H1	531	1 (Reference)				
H2	119	4.14 (2.57–6.63)	< 0.001		1.06 (0.56–2.00)	0.849
H3	1,074	1.19 (0.86–1.67)	0.307		1.38 (0.93–2.07)	0.110
H6	28	4.71 (2.01–10.54)	0.001		2.93 (0.96–8.22)	0.048
H7	304	0.89 (0.55–1.42)	0.644		1.02 (0.56–1.84)	0.937
H8	363	1.82 (1.23–2.68)	0.003		1.37 (0.86–2.20)	0.186
H10	185	1.09 (0.63–1.82)	0.761		0.56 (0.29–1.06)	0.082
H11	104	3.28 (1.95–5.44)	< 0.001		1.77 (0.89–3.45)	0.100
H12	17	4.63 (1.54–12.66)	0.004		1.58 (0.42–5.29)	0.473
H13	150	1.16 (0.64–2.00)	0.614		1.30 (0.65–2.49)	0.447

CI = confidence interval; OR = odds ratio; AVPU = alert, verbal response, pain response and unresponsive.
